# Exposure to Domestic Violence During the COVID-19 Pandemic: A Potent Threat to the Mental Well-being of Children

**DOI:** 10.21315/mjms2021.28.3.16

**Published:** 2021-06-30

**Authors:** Woi Hon Boo

**Affiliations:** Ampang Baru Community Clinic, Ipoh, Perak, Malaysia

**Keywords:** coronavirus disease 2019, COVID-19, mental, health, well-being, children, abuse, domestic, violence

## Abstract

The unprecedented scale of the coronavirus disease 2019 (COVID-19) pandemic has indeed brought about tremendous ramifications on the state of mental health for children. One notable aspect deserving emphasis however, is the psychological impact in children as a result of the purported surge of domestic violence (DV) in many of the countries that imposed stay-at-home requirement. The imposition of movement restriction policy led to isolation and overcrowding, made worse by loss of income in some instances, causing high levels of stress and anxiety, thereby increasing the risk of DV and child abuse particularly those already living within violent or dysfunctional families. Children exposed to DV have higher risk of developmental delay and behavioural problems, more likely to suffer from post-traumatic symptoms, depression and anxiety, reduced cognitive functioning and social competence. Additionally, these children tend to have poorer academic performance and are more likely to engage in violent or abusive relationship later in life. Health care providers need to be vigilant and actively screen and identify children or families that are affected by DV to protect their physical and mental health well-being.

Dear Editor,

I read with interest the recent article by Ramadhan et al. where they discussed how the unprecedented scale of the coronavirus disease 2019 (COVID-19) pandemic has brought about tremendous ramifications on the state of children’s mental health (1). Apart from the points raised in the article, one notable aspect deserving emphasis is the psychological impact in children as a result of the purported surge of domestic violence (DV) in many of the countries that imposed a stay-at-home requirement (2, 3). This observation is consistent with prior studies which reported that during times of health crises, such as the Ebola and Zika virus outbreaks, DV incidence tends to surge (3).

The imposition of a movement restriction policy led to isolation and overcrowding, made worse by loss of income in some instances. This situation caused high levels of stress and anxiety, thereby increasing the risk of DV and child abuse, particularly in those already living within violent or dysfunctional families. This is aggravated further by the increased barrier in accessing normal social support and family or child protection services due to shelter-in-place orders, which inadvertently confined the victims with their abusers (2, 4).

Exposure to DV has detrimental effects on the mental health of children. Children exposed to DV are at a higher risk of developmental delay and behavioural problems; they are more likely to suffer from post-traumatic symptoms, depression and anxiety and reduced cognitive functioning and social competence. These children also tend to have poorer academic performance and are more likely to engage in violent or abusive relationships later in life (5).

The disruption of normal child protection and response services in most countries due to the current pandemic underscores the urgent need for health care providers at all levels, be it primary care, emergency departments or specialised tertiary care especially the paediatric and psychiatric services to be vigilant and actively screen and identify children or families who are affected by DV. If DV is suspected, crisis intervention should be initiated to protect not only the physical safety of these children but also their emotional and mental well-being.
